# Mine drainage leads to the reshaping of the spatial patterns of soil extracellular econzymatic stoichiometry and microbial resource limitation

**DOI:** 10.1038/s41598-025-20858-1

**Published:** 2025-11-27

**Authors:** Ruoshi Ma, Jieru Kong, Hongxia Mou, Bingru Liu

**Affiliations:** 1 School of Biological Science and Engineering, North Minzu University, Yinchuan, 750021 China; 2 School of Ecological Environment, Ningxia University, Yinchuan, 750021 China

**Keywords:** Mine water, Desert grasslands, Ecoenzymatic stoichiometry, Microbial resource limitation, Ecology, Biogeochemistry, Ecology, Environmental sciences

## Abstract

Global environmental changes have brought significant attention to the ecological problems caused by mine drainage. Mine drainage alters the physicochemical properties of soil, which can, in turn, influence its ecological function. However, the impact of mine drainage on soil ecoenzymatic carbon–nitrogen-phosphorus (C-N-P) stoichiometry and the spatial distribution of microbial resource limitations(i.e., carbon, nitrogen, or phosphorus limitation) across various soil depths and distances from discharge points is not well understood. Therefore, we investigated the variations in physicochemical properties, microbial biomass, extracellular enzyme activities, and ecoenzymatic stoichiometry of soils at varying distances and depths surrounding mine water discharge areas. Furthermore, microbial resource limitations were measured through vector analysis of ecoenzymatic stoichiometry. The results showed that mine drainage significantly enhanced soil extracellular enzyme activity. While the discharge had no substantial impact on ecoenzymatic stoichiometry across different distances, significant changes were observed at various soil depths. Specifically, ecoenzymatic stoichiometry decreased consistently with increasing soil depth. spatial distribution of microbial resource limitations in the study area were predominantly phosphorus-limited, with P limitation intensifying with greater soil depth. Overall, these findings improve our knowledge of how mine drainage influences soil ecoenzymatic stoichiometry and the spatial distribution of microbial resource constraints, providing insights for future ecological management strategies.

**Supplementary Information:**

The online version contains supplementary material available at 10.1038/s41598-025-20858-1.

## Introduction

Most of China’s coal resources are concentrated in the arid regions of northwestern China. With ongoing economic growth, coal mining intensity has steadily increased, leading to a corresponding rise in mine drainage, now a major environmental concern. Mine water contains high concentrations of pollutants, including nitrogen, phosphorus, sulfates, and chlorides^1,2^. The release of these pollutants into the environment directly affects ecosystems and alters the physicochemical properties of surrounding soils^3^. This pollution exacerbates soil degradation, destabilizes fragile ecosystems like desert grasslands, accelerates soil salinization, and leads to shifts in vegetation types and species. Changes in vegetation types directly or indirectly impact soil’s physical structure, nutrient cycling, microbial biomass, microbial community diversity, and extracellular enzyme activity. Studies have shown that factors such as soil moisture, salinity, nutrient availability, pH, and microbial biomass significantly influence soil enzyme activity^4–8^, which plays a crucial role in material cycling within desert grassland ecosystems.

Soil microbial biomass and enzyme activity respond rapidly to environmental changes and external disturbances. Microbial biomass plays a vital role in nutrient cycling, organic matter storage, pathogen suppression, residue decomposition, and pollutant degradation. It also serves as a source of soil enzymes^9^ .,. Soil enzymes, which are regulated by environmental nutrient availability, serve as indicators of biodiversity, ecosystem function, and soil fertility^10^. Researchers have increasingly focused on extracellular enzyme activities, particularly those related to carbon (β-glucosidase, BG), nitrogen (β-N-acetylamino glucosidase, NAG, leucine aminopeptidase, LAP), and phosphorus (alkaline phosphatase, ALP) cycles. These enzymes are closely associated with microbial limitation and biochemical processes, reflecting microbial nutrient demand^11,12^ . For example, β-glucosidase plays a critical role in terrestrial carbon cycling by catalyzing the final step in cellulose degradation, providing an essential energy source for microbial biomass^13^. NAG is involved in the degradation of chitin and indicates nitrogen uptake by plants, while LAP breaks down hydrophobic amino acids to release inorganic nitrogen for plant growth and participates in soil nitrogen cycling^14^. ALP, on the other hand, converts organic phosphorus into bioavailable forms and indicates soil organic phosphorus mineralization potential^15–17^. Previous studies found that BG, AP, NAG, and LAP showed positive correlations with soil nutrients (Total Carbon and Total Nitrogen contents) and negative correlations with MAT (Mean Annual Temperature), clay content, and BD bulk density (Bulk Density)^18^. Dong et.al.^19^ also found that soil nutrients can regulate enzyme activities by influencing the available substrates and nutrient stoichiometry. The stoichiometric ratios of soil enzymes not only reveal microbial nutrient demands and limitations but also reflect the relative rates of soil carbon, nitrogen, and phosphorus cycling. This enables researchers to assess the balance between microbial metabolism, nutrient demand, and environmental nutrient availability^20,21^. Two widely used models for estimating microbial metabolic limitations are the threshold element ratio (TER) model and the enzyme allocation model^11,22–24^. Although TER cannot pinpoint the single most limiting nutrient, the enzyme allocation model, developed by Moorhead et al.^22^, quantifies carbon limitation using vector length and nitrogen or phosphorus limitation using vector angles. The vector model quantifies microbial acquisition of C, N, and P simultaneously by plotting the allocation proportions of enzyme activities to generate vector lengths and angles. It is based on simplified empirical evidence that the C:N:P enzyme activity ratio is approximately 1:1:1 at the global scale^11,12,25^. One of the advantages of the vector model is that it is not affected by individual enzymes and can reflect the overall relative resource (C, N, and P) demands of microbial metabolism. In addition, it can identify whether microbial metabolism in a specific environment is limited by N or P, rather than being co-limited by both. This model provides a holistic view of microbial resource requirements and identifies the most limiting nutrient in a given environment. Considering the crucial role of soil microorganisms in maintaining ecosystem multifunctionality (e.g., plant diversity, nutrient cycling, and retention)^26^, understanding microbial resource limitation patterns and their driving factors is essential for effective management strategies in desert grasslands. Previous studies have analyzed the limitations of microbial metabolism in different ecosystems (e.g., forests, grasslands, and farmlands) based on soil enzyme stoichiometry^27–29^.

Previous studies have shown that mine water drainage significantly influences sucrase, dehydrogenase, and ALP levels in surrounding soils^30,31^. Soil physicochemical properties vary spatially around mine water discharge points, which can lead to reduced urease and sucrase activities^32^. Sheng et al.^33^ found that in aerobic conditions, iron—bearing minerals like nontronite and magnetite, with structural Fe(II), can generate reactive oxygen species (ROS), especially hydroxyl radicals, through oxidation. These ROS can severely damage the structure of extracellular enzymes such as β—glucosidase, significantly reducing their activity and lifespan. The production of hydroxyl radicals on the mineral surface is inversely proportional to pH and directly proportional to the oxidation degree of structural Fe(II). Dong et al.^34^ found the coupling between iron mineral redox cycling and organic matter transformation. ROS from Fe(II) oxidation can either decrease or increase extracellular enzyme activity and microbial activity. Sheng et al.^35^ reported that the adsorption capacity of soil minerals, determined by properties like specific surface area (SSA), plays a key role in regulating enzyme activity. Minerals with high SSA can adsorb enzymes, leading to conformational changes and reduced substrate encounter probability, ultimately inhibiting enzyme activity. Du et al.^36^ indicated that nitrogen (N) limitation predominates in tundra, boreal forests, temperate coniferous forests, montane grasslands, and shrublands. In contrast, phosphorus (P) limitation is more prevalent in tropical and subtropical forests, temperate broadleaf forests, deserts, Mediterranean vegetation, as well as tropical, subtropical, and temperate grasslands, savannas, and shrublands. Despite the known impacts of mining on soil and water quality, there is limited understanding of how mine drainage affects the spatial distribution of microbial enzyme activities and resource limitations across different soil depths and distances from discharge points. To address this gap, we investigated desert grassland sites near water drainage areas from four coal mines in Ningxia Province, China. We hypothesize (H1) that extracellular enzyme activity is higher in shallow soils (0–10 cm) closer to the mining water discharge point, while its influence diminishes in deeper soil layers (20–30 cm) further to the mining water discharge point. Furthermore, we propose (H2) that soil microorganisms in the study area are primarily constrained by carbon and phosphorus, with notable differences in microbial resource limitations across different soil depths. Specifically, shallow soils are more prone to carbon limitation, whereas deeper soils are more likely to experience phosphorus limitation.

## Results

### The spatial variation in soil physiochemical properties and microbial biomass

**Fig. 1:**
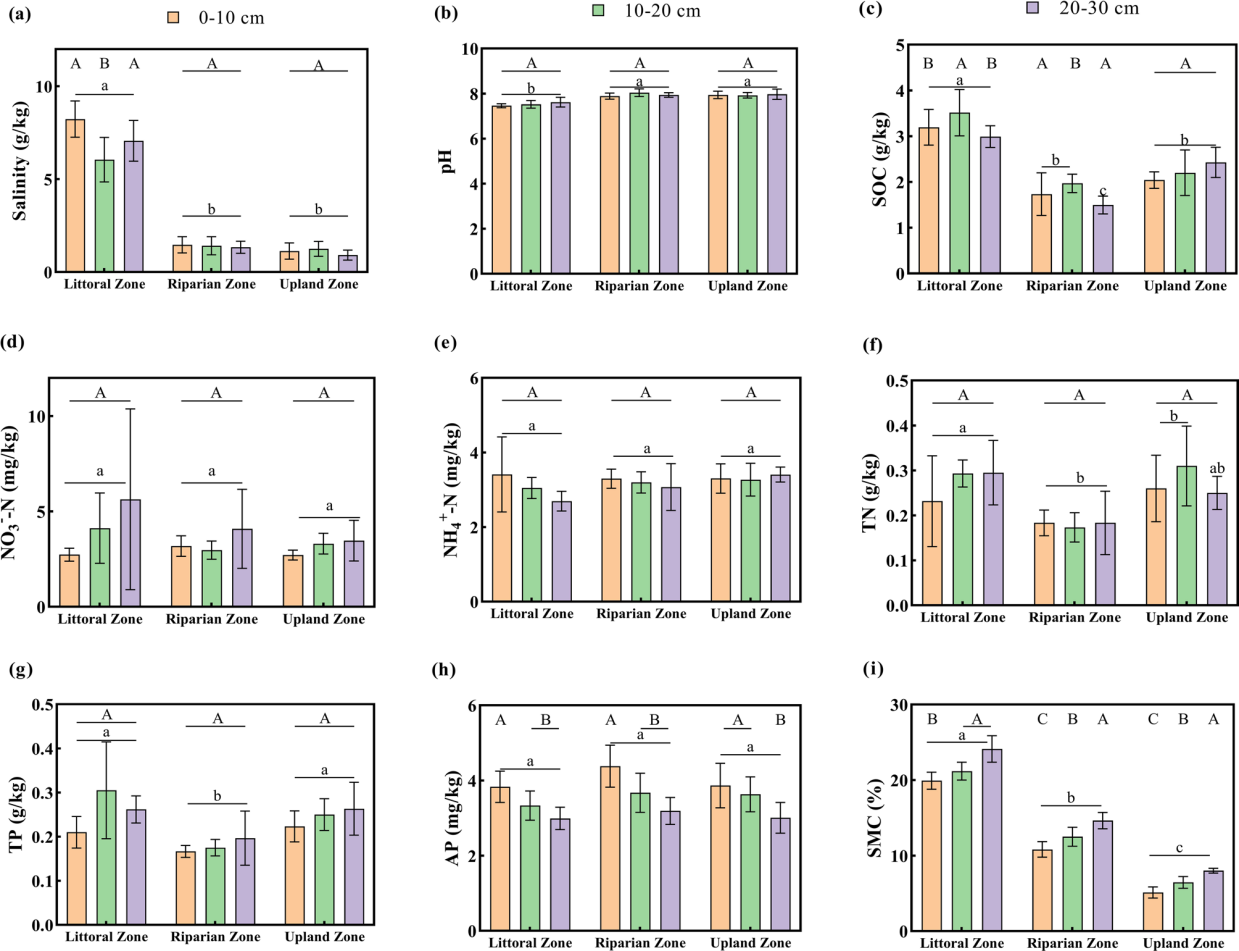
Soil physiochemical properties in the three zones and three soil layers. SOC—soil organic carbon; NH_4_^+^-N—ammonium nitrogen; NO_3_^-^-N—nitrate nitrogen; TN—total nitrogen; TP—total phosphorus; AP—available phosphorus; and SMC—soil moisture content. Vertical lines represent the standard error (SE) of the mean (n = 6), indicating the variability of replicate measurements. Letters above the bars indicate significant differences (p < 0.05) based on one-way ANOVA followed by Tukey’s HSD post-hoc test. Capital letters (A, B, C) denote significant differences between different soil depth within the same zone. Lowercase letters (a, b, c) denote significant differences between different zones within the same soil layer. The same applies to all of the figures and tables.

Mine drainage significantly altered soil pH, SMC, SOC, TP, and TN (Fig. 1, *P* < 0.05). The soil pH was notably lower in the littoral zone compared to the riparian and upland zones (*P* < 0.05). SMC decreases with increasing distance from the mine drainage point. For the 0–10 cm and 10–20 cm soil layers, SOC was significantly higher in the littoral zone than in the riparian and upland zones (*P* < 0.05). Similarly, TN and TP contents were significantly higher in the littoral and upland zones compared to the riparian zone (*P* < 0.05). In contrast, mine drainage had minor, insignificant effects on AP, NO₃⁻-N, and NH₄⁺-N across the three zones (*P* > 0.05). SMC, SOC, and AP showed significant variation across soil layers (Fig. 1). In the littoral and riparian zones, SOC was significantly higher in the 10–20 cm layer compared to the 20–30 cm layer, with no significant difference between the 0–10 cm and 10–20 cm layers. AP content significantly decreased with increasing soil depth in all zones (*P* < 0.05), while SMC significantly increased with depth. pH, TN, TP, NO₃⁻-N, and NH₄⁺-N showed minimal variation among soil layers and did not significantly change with increasing depth in any zone (*P* > 0.05). Two-way ANOVA revealed that the interaction between mining distance and soil depth had a significant effect on soil salinity in the study area (*P* = 0.002; Table S2).

**Fig. 2:**
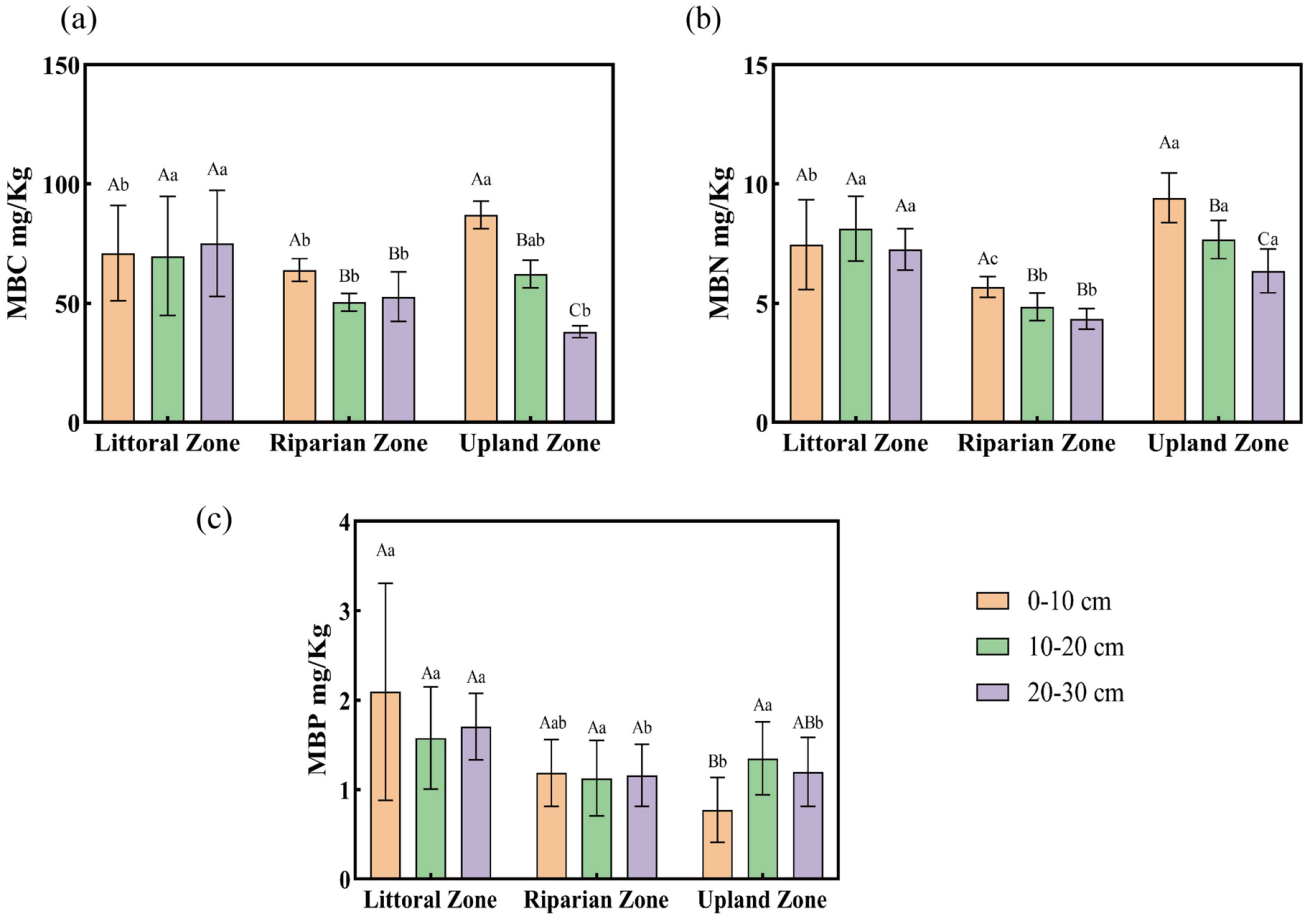
Soil microbial biomass in the three zones and three soil layers (mean ± S.D.). (**a–c**): microbial biomass carbon (MBC), microbial biomass nitrogen (MBN), and microbial biomass phosphorus (MBP), respectively.

The mine drainage had varying impacts on the soil MBC, MBN, and MBP contents (Fig. 2). In the 0–10 cm layer, MBC was lower in the riparian and nearshore areas than in the natural zone, while in deeper layers (10–20 cm and 20–30 cm), MBC was higher in the riparian zone (Fig. 2a). MBN was lower in the littoral and riparian zones at 0–10 cm, but lower in the riparian zone at deeper layers (Fig. 2b). MBP was higher in the littoral zone at 0–10 cm and 20–30 cm compared to other zones (Fig. 2c). Across the littoral zone, there were no significant changes (*P* > 0.05) in MBC, MBN, or MBP contents with increasing soil depth (Fig. 2). However, in the riparian and upland zones, both MBC and MBN decreased with increasing depth (*P* < 0.05; Figs. 2a,b). MBP content in the littoral and riparian zones did not significantly vary with depth (*P* > 0.05), while in the upland zone, it peaked in the 10–20 cm layer (Fig. 2c). ANOVA revealed significant interactions between zone type and soil depth for MBC (*P* = 0.000) and MBN (*P* = 0.013) (Table S2).

### The spatial distribution in soil extracellular enzyme activities.

**Fig. 3:**
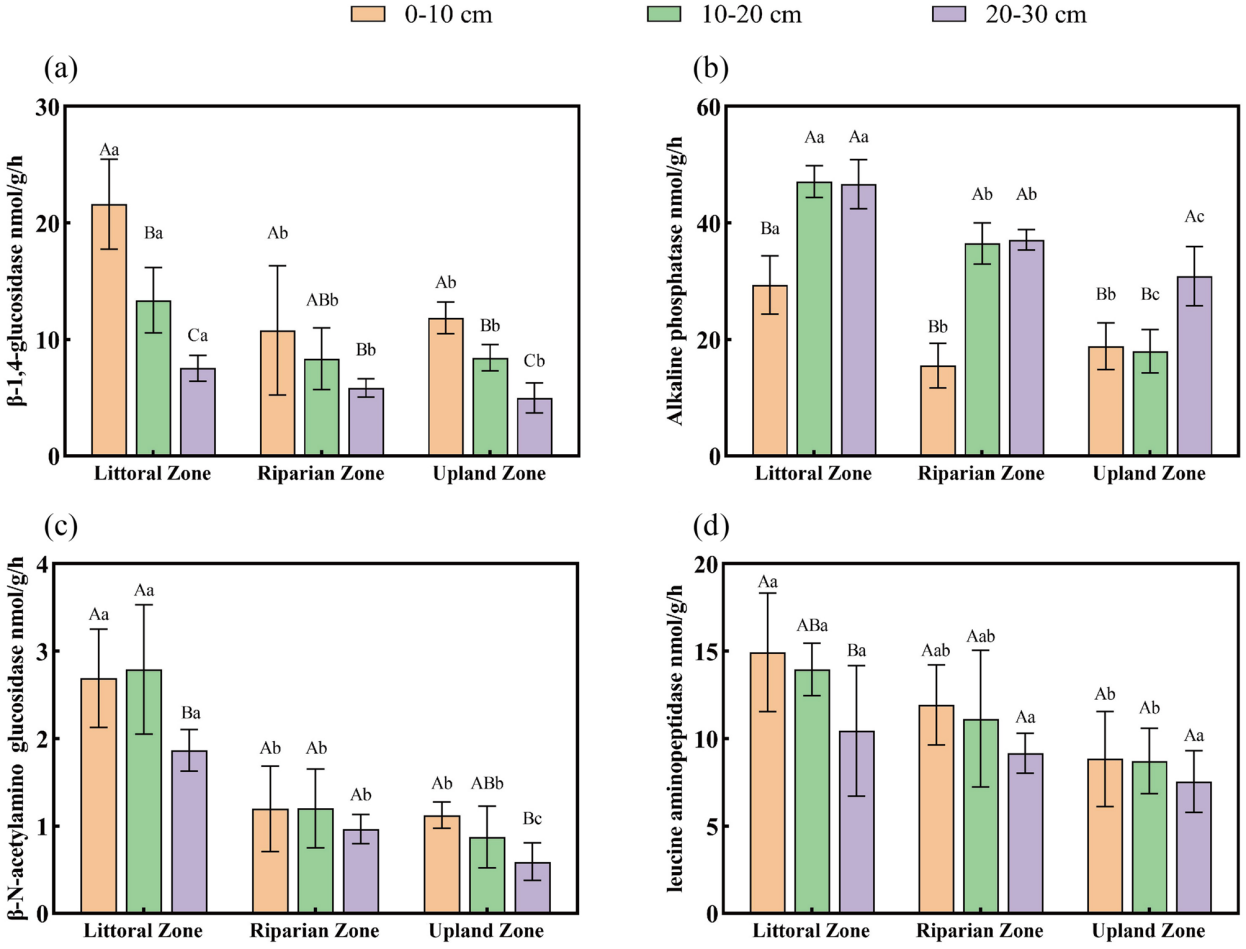
Soil extracellular enzyme activities in the three zones and three soil layers (mean ± S.D.). (**a**–**d**) β-1,4-glucosidase (BG); alkaline phosphatase (ALP); β-N-acetylamino glucosidase (NAG); and leucine aminopeptidase (LAP), respectively.

Mine drainage significantly altered the activities of BG, ALP, NAG, and LAP in the 0–30 cm soil layer (Fig. 3). Across the 0–30 cm layer, BG, NAG, and LAP activities were consistently highest in the littoral zone, with significant differences compared to the riparian and upland zones (*P* < 0.05; Fig. 3a,c,d). Vertically, BG activity decreased significantly with depth across all zones (*P* < 0.05), while LAP activity decreased with depth only in the littoral zone (*P* < 0.05) and remained stable in the riparian and upland zones across all depths (*P* > 0.05; Fig. 3d). ALP activity showed the opposite vertical trend in the 0–30 cm layer: it increased significantly with depth in all zones (*P* < 0.05) and was consistently higher in the littoral zone than in the riparian and upland zones at the same depth (*P* < 0.01; Fig. 3b). Within the 10–20 cm and 20–30 cm layers specifically, ALP activity was also significantly higher in the riparian zone than in the upland zone (*P* < 0.05; Fig. 3b). For NAG, in addition to the overall higher activity in the littoral zone across 0–30 cm, significant differences between the riparian and upland zones were observed at the 20–30 cm depth (*P* < 0.05; Fig. 3c).

Two-way ANOVA showed significant interactions between zone type and soil depth for BG (*P* = 0.003; Table S2) and ALP activities (*P* = 0.000; Table S2).

### The spatial pattern in soil extracellular ecoenzymatic stoichiometry and microbial resource limitation

**Fig. 4:**
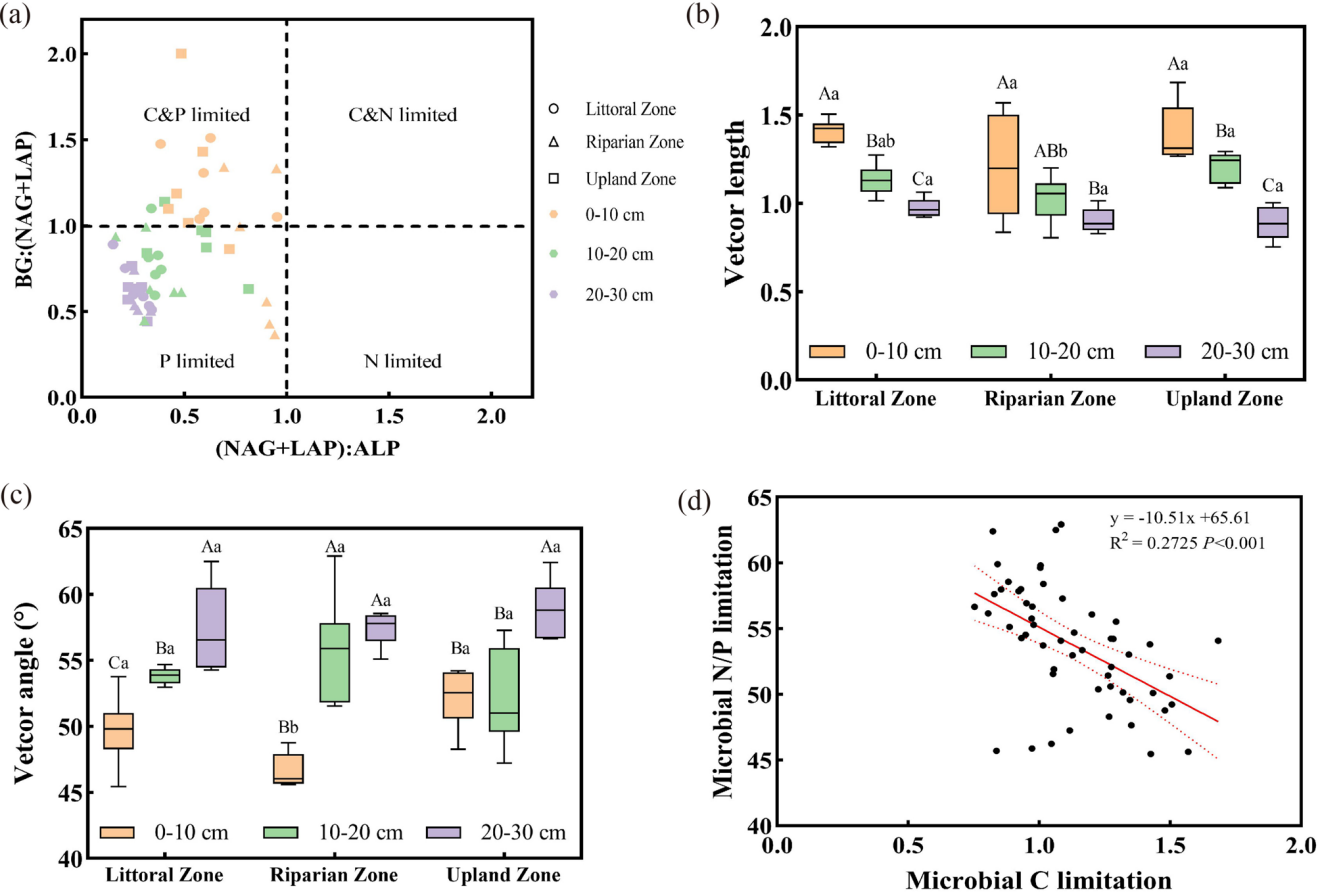
(**a**): Scatter plots of soil ecoenzymatic stoichiometry among different zones and different soil layers, (**b**): Soil microbial C limitation, quantified by vector length (derived from the enzyme vector model). Vector length is calculated as the Euclidean distance from the origin in a 2D space defined by ln(BG/ALP) (x-axis) and ln[BG/(NAG + LAP)] (y-axis), where longer lengths indicate stronger C limitation. (**c**): Soil microbial N/P limitation, quantified by vector angle (derived from the enzyme vector model). Vector angle is calculated as the arctangent of [ln(BG/ALP)/ln(BG/(NAG + LAP))], where angles < 45° indicate N limitation and angles > 45° indicate P limitation (**d**) linear regression relationship between soil microbial C limitation and microbial N/P limitation. The soil microbial C limitation is represented by the vector length, while the soil microbial N/P limitation is represented by the vector angle. Angles of < 45° indicate N limitation, and angles of > 45° indicate P limitation.

Mine drainage significantly affected the stoichiometric vector lengths and angles of soil extracellular enzymes, as well as the enzymatic N/P ratio (E_N:P_) and C/P ratio (E_C:P_) (*P* < 0.05), but did not influence the enzymatic C/N ratio (E_C:N_) (*P* > 0.05) (Fig. 4, Table 1). Vector lengths were shorter in the 10–20 cm soil layer of the littoral and riparian zones compared to the upland zone (Fig. 4b), while changes in vector angles with depth varied across zones (Fig. 4c). The E_N:P_ ratio was significantly lower in the littoral and riparian zones than in the upland zone at 10–20 cm, but significantly higher in the riparian zone at 0–10 cm. Similarly, the E_C:P_ ratio was significantly lower in the littoral and riparian zones than in the upland zone at 10–20 cm (Table 1). Significant differences in E_N:P_, E_C:N_, and E_C:P_ ratios, as well as vector lengths and angles, were observed across soil layers (*P* < 0.05) (Table 1). Both E_N:P_ and E_C:P_ ratios declined with increasing depth.

**Table 1:** Variations characteristics of the extracellular ecoenzymatic stoichiometric ratios.

Zone	Soil layer	E_C:N_	E_C:P_	E_N:P_
Littoral zone	0–10 cm	1.24 ± 0.22^Aa^	0.75 ± 0.19^Aa^	0.62 ± 0.18^Ab^
	10–20 cm	0.80 ± 0.17^Ba^	0.28 ± 0.05^Bb^	0.36 ± 0.02^Bb^
	20–30 cm	0.65 ± 0.15^Ba^	0.16 ± 0.02^Ba^	0.26 ± 0.07^Ba^
Riparian zone	0–10 cm	0.84 ± 0.45^Aa^	0.70 ± 0.36^Aa^	0.86 ± 0.10^Aa^
	10–20 cm	0.71 ± 0.21^Aa^	0.23 ± 0.07^Bb^	0.34 ± 0.11^Bb^
	20–30 cm	0.58 ± 0.09^Aa^	0.16 ± 0.02^Ba^	0.27 ± 0.03^Ba^
Upland zone	0–10 cm	1.27 ± 0.41^Aa^	0.66 ± 0.20^Aa^	0.53 ± 0.11^Ab^
	10–20 cm	0.90 ± 0.17^Ba^	0.49 ± 0.12^Ba^	0.55 ± 0.17^Aa^
	20–30 cm	0.62 ± 0.11^Ba^	0.16 ± 0.03^Ca^	0.26 ± 0.04^Ba^

E_C:N_—ln BG/ln (NAG + LAP); E_C:P_ -ln BG/ln AP; E_N:P_—ln (NAG + LAP)/ln AP. The data in the tables are the mean ± standard error, and the differences are considered to be significant at P < 0.05.

Enzyme vector model analysis of nutrient utilization efficiency quantified the ecoenzymatic stoichiometry in the study area (Fig. 4). In study areas, soil microorganisms were co-limited by C and P (Fig. 4a). Vector length decreased with soil depth, suggesting reduced carbon limitation, while vector angles increased, reaching a maximum at 20–30 cm, indicating heightened phosphorus limitation (Fig. 4b,c). Furthermore, microbial C limitation negatively correlated with microbial N/P limitation (*P* < 0.001) (Fig. 4d).

### Parameters controlling soil extracellular ecoenzymatic stoichiometry and microbial resource limitation

**Fig. 5:**
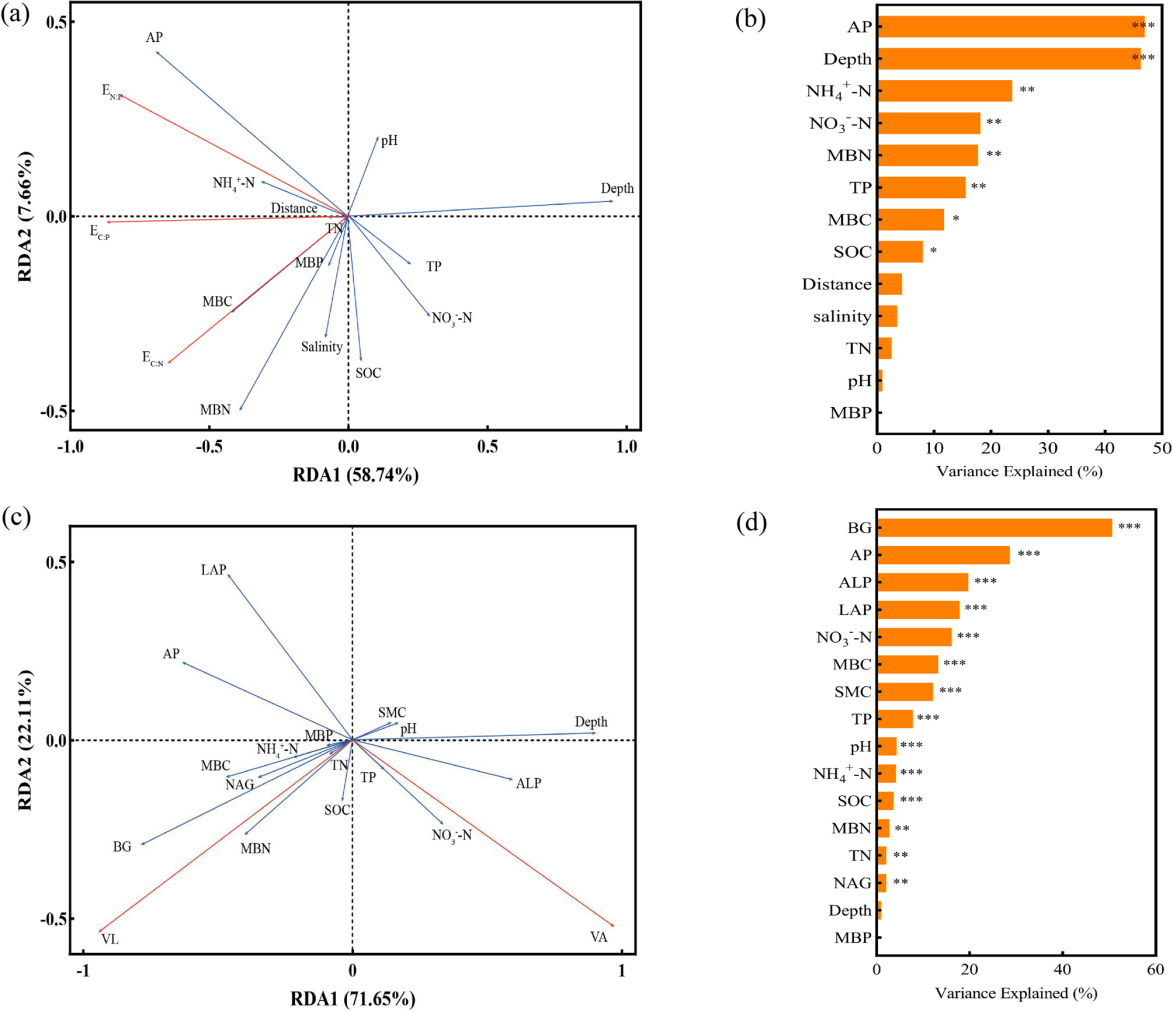
Redundancy analysis of the soil abiotic and biotic factors effect on enzyme activity and ecoenzymatic stoichiometry (**a**) and microbial resource limitation (**c**) .Envfit analysis identified key factors of enzyme activity and ecoenzymatic stoichiometry (**b**) and microbial resource limitation (**d**). Significance levels are as follows: *p < 0.05;**p < 0.01; ***p < 0.001.

According to the detrended correspondence analysis (DCA), the gradient length of the sorting axis (LGA) was less than 3, indicating that the use of RDA to analyze the relationships between the soil ecoenzymatic stoichiometry and microbial resource limitation and the soil physicochemical factors and microbial biomass is reasonable (Fig. 5a,c). The first and second axes of the model explained 41.58 and 24.19% of the variation in the enzyme activity and ecoenzymatic stoichiometry, and 83.02 and 16.11% of the variations in the vector length and angle, respectively (Fig. 5a,c). The results of the RDA and envfit analysis indicated that in the study area, the ecoenzymatic stoichiometry were positively correlated with MBC, MBN, AP and NH_4_^+^-N, but negatively correlated with NO_3_^-^-N. A strong negative correlation was observed between ecoenzymatic stoichiometry and soil depth (Fig. 5a, b). Furthermore, shifts in soil microbial P limitation were strongly positively correlated with ALP, SMC, SOC, NO_3_^-^-N, mining distance and soil depth, and negatively correlated with AP, LAP, and E_N:P_ (Fig. 5c,d). Additionally, soil microbial C limitation was positively associated with BG, NAG, MBN, E_C:P_ and E_C:N_, while negatively associated with soil depth, pH, and SMC (Fig. 5c,d).

**Fig. 6:**
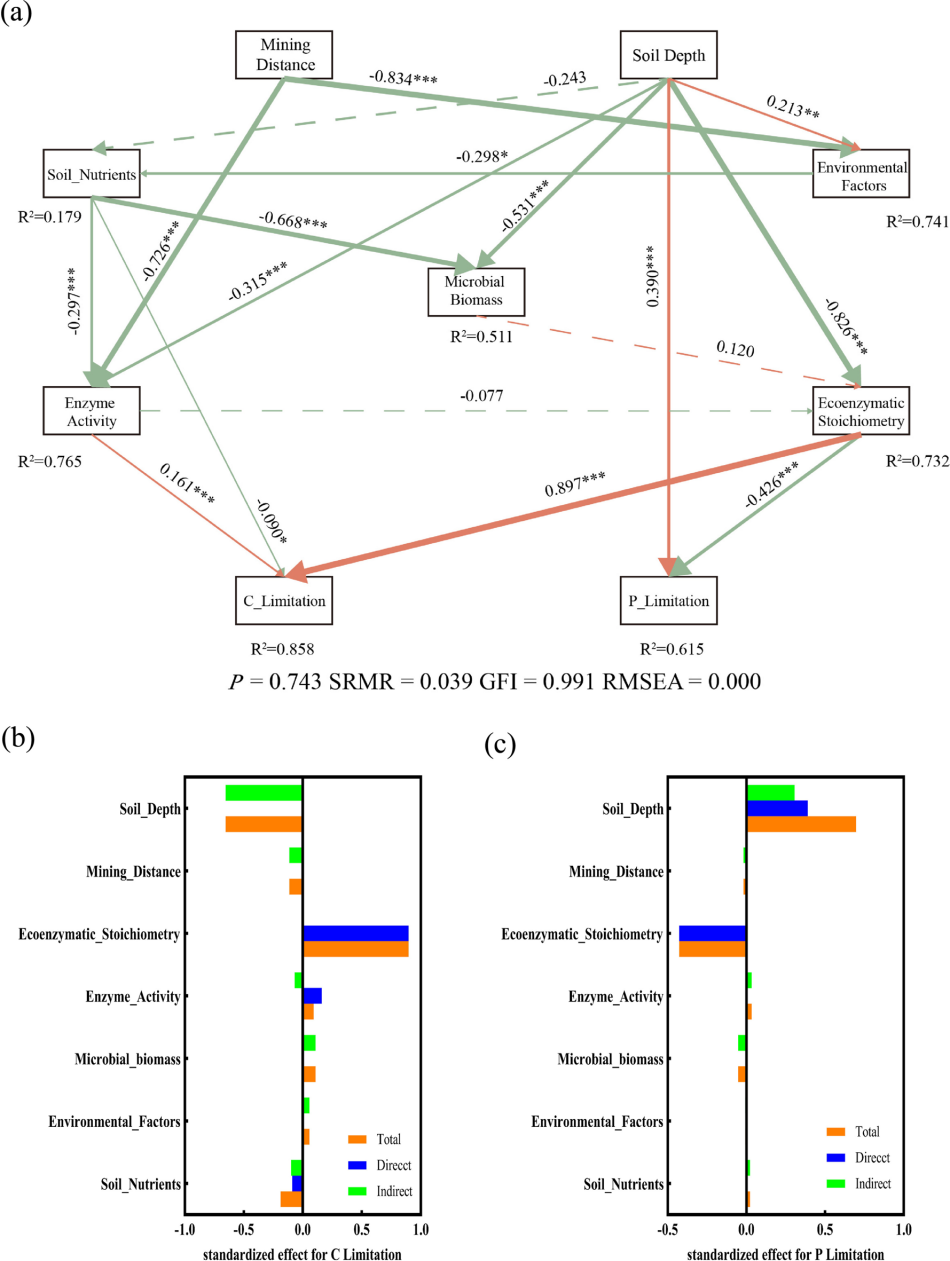
Partial least squares structural equation modeling (PLS-SEM) showed the direct and indirect effects of mining distance, soil depth, soil nutrients, environmental factors and microbial biomass on C limitation (a)and P limitation (c). The circles represent the r^2^ of each variable in the model, indicating the extent to which the model explains the variable, and the lines represent the paths connecting the variables. The numbers associated with each line are the standardized path coefficients. The solid lines denote significant paths (*P* < 0.05), the dashed arrows denote non-significant paths, orange-red indicates positive correlation paths, and green indicates negative correlation paths.; ***P < 0.001, **P < 0.01, *P < 0.05. (b and c) standardized total effects (STE) of each factor on the C limitation and P limitation. Soil Nutrients (integrating AP, NO_3_^-^-N), Microbial Biomass (integrating MBC, MBN), Enzyme Activity (integrating BG, LAP, ALP), Ecoenzymatic Stoichiometry (integrating E_C:N_, E_C:P_, E_N:P_).

To clarify the direct and indirect effects of soil environmental factors and biological factors on soil ecoenzymatic stoichiometry and microbial resource limitation, we screened environmental factors with significant impacts on microbial resource limitation based on the results of RDA and envfit analyses, integrated them into composite variables, and then used these variables to construct the PLS-SEM. (Fig. 6a). The model exhibited a good fit to the data (P_Limitation, NFI = 0.982, SRMR = 0.031; C_Limitation, NFI = 0.968, SRMR = 0.027), with R^2^ values of 0.703 and 0.875 for explaining P and C limitation, respectively. By applying PLS-SEM and computing the total standardized effects of each factor on microbial carbon and phosphorus limitations, we find that the mining distance had a negative effect on C_limitation by affecting enzyme activity (std.all = 0.674, *P* < 0.001). Soil depth exerted a negative effect on C_limitation through its influences on microbial biomass (std.all = 0.415, *P* < 0.001), soil nutrients (std.all = 0.622, *P* < 0.001), enzyme activity (std.all = 0.227, *P* = 0.015), and ecoenzymatic stoichiometry (std.all = 0.844, *P* < 0.001). The standardized total effects indicated that soil nutrients were the only variable with a positive impact on C_limitation. Ecoenzymatic stoichiometry and soil depth, which both had significant negative effects, were the two most influential factors on C_limitation. The mining distance significantly positively affected P_limitation by prominently influencing enzyme activity (std.all = 0.674, *P* < 0.001). In contrast, soil depth impacted PL mainly through its significant effects on soil nutrients (std.all = 0.622, *P* < 0.001) and ecoenzymatic stoichiometry (std.all = 0.844, *P* < 0.001). All variables exerted positive effects on P_limitation, with soil depth being the most influential factor, followed by ecoenzymatic stoichiometry and soil nutrients.

## Discussion

### Impact of the mine drainage on the soil physicochemical properties and the microbial biomass

In the study area, soil salinity, moisture content, and organic carbon levels were significantly higher in the littoral zone compared to the riparian and upland zones. This is likely due to the littoral zone’s proximity to the mine water source, which features high flowability and permeability. The sustained or intermittent release of mine water creates a hydraulic gradient, prompting the migration of salt-rich mine water towards the littoral zone. Additionally, the soil’s porosity and permeability govern the vertical and horizontal transport of salt ions with water. In the littoral zone, prolonged exposure to mine water flow leads to the absorption or retention of salt ions by soil particles as water percolates, resulting in salt accumulation at the surface^37,38^. Additionally, the riparian zone acted as a retention area for certain salt ions (e.g., SO_4_^2-^ and Cl^-^) from the mine water. The elevated soil salinity in the littoral zone (Table S1), along with the presence of salt-tolerant plants, contributed to an increase in soil organic carbon content^39^. Research indicates that the cultivation of Suaeda salsa leads to an increase in soil organic matter, particularly in the top 0–20 cm of soil^40^. The decomposition of dead plant material, the presence of residual roots, and the secretion of organic compounds by roots all contribute to the enhancement of soil organic matter. Salt-tolerant plants introduce organic carbon into the soil through root exudates^41^. These exudates not only help in adjusting the rhizosphere pH to mitigate salt stress but also serve as substrates for microorganisms, facilitating the synthesis and stabilization of carbon derived from microbial activity. Additionally, the increased microbial population in the rhizosphere, coupled with enhanced metabolic activity, results in a higher availability of nutrients in the soil for plant uptake^42^. In the riparian and upland zones, the highest levels of MBC and MBN were found in the 0–10 cm soil layer. This is attributed to the influence of above-ground vegetation in these areas, which led to greater accumulation of humus and litter, providing a favorable environment for microbial growth and reproduction through root exudates and litter decomposition. In contrast, MBC and MBN did not show significant variation across different soil layers in the littoral zone. This lack of variation may be due to the proximity to the mine water drainage site, soil compaction, sparse vegetation, and exposure of the surface soil, which resulted in reduced MBC and MBN sources.

### Influence of the mine drainage on the soil extracellular enzyme activity and its stoichiometry

The activity of BG decreased with increasing soil depth across all zones, being significantly higher in the 0–10 cm layer compared to the 20–30 cm layer, consistent with the findings of Li et al.^43^ In contrast, ALP activity increased with soil depth and was significantly higher in the 20–30 cm layer than in the 0–10 cm layer. The vertical distribution pattern of BG activity can be attributed to: (1) the decomposition of litter in surface soil layers, facilitated by high air permeability, which provided nutrients such as organic carbon that promoted microbial growth and thus higher SOC content; (2) SOC serves as a crucial carbon source for soil microorganisms, while BG plays a key role in the carbon cycle. As SOC levels increase, the demand for carbohydrate decomposition by microorganisms is stimulated, leading to increased BG production and, consequently, enhanced BG activity^44^. According to resource allocation theory, Allison et al.^45^ demonstrated that when microbial growth is limited by a specific element, the activity of extracellular enzymes associated with that element increases. As microbial growth in the study area was primarily limited by phosphorus (P), ALP activity increased, leading to higher ALP levels in the soils. This increase in ALP activity alleviated microbial phosphorus limitation, aligning with Liu et al.^46^.

In all three soil layers, activities of BG, ALP, NAG, and LAP were highest in the littoral zone and lower in the riparian and upland zones, consistent with our first hypothesis. This phenomenon can be attributed to: (1) Soil moisture affecting the diffusion of substrates, enzymes, and their products; drought conditions limit enzyme and substrate diffusion^47,48^. Soil moisture content was significantly higher in the littoral zone compared to the riparian and upland zones, and other studies have shown a significant positive correlation between soil moisture content and extracellular enzyme activity^46^; (2) higher soil salinity in the littoral zone promoted the growth of salinity-tolerant plants, increasing the abundance of halophytes and thereby the total amount of soil microorganisms in the littoral zone^39^, which in turn enhanced soil enzyme activities. Therefore, all four extracellular enzymes exhibited the highest activity levels in the littoral zone. The elevated enzyme activity in the litttoral zone (Fig. 3) is not only linked to high soil moisture facilitating substrate diffusion but also likely associated with the capacity of minerals to adsorb enzymes. Previous studies have demonstrated the influence of specific surface area (SSA) on enzyme activity — in high-SSA mineral-rich soils, enzyme adsorption reduces instantaneous activity but enhances functional longevity^35^. Fine-particle minerals from mine drainage likely enhance specific surface area (SSA) locally in riparian zone soils. This SSA elevation maintains long-term extracellular enzyme activity through strong adsorption (Fig. 3). Conversely, upland zone soils experience minimal mine drainage influence and consist predominantly of sandy textures with high sand content. Their low SSA constrains mineral adsorption capacity for enzymes, resulting in accelerated enzymatic activity decay.

The ratios of E_C:N_, E_N:P_, and E_C:P_ decreased with soil depth, indicating that while the activities of both nitrogen- and phosphorus-acquiring enzymes increased, phosphorus-acquiring enzyme activity was higher. The average soil enzymatic C:N and C:P ratios were 0.84 and 0.40, respectively, both lower than the global averages of 1.14 and 0.62. The average soil enzymatic N:P ratio was 0.45 (global average: 0.44)^49^. The high activity of soil nitrogen-cycling enzymes and ALP under mine drainage disturbance suggests that local soils were mainly phosphorus-limited and relatively nitrogen-deficient, consistent with Yu et al.^32^and the observation that alkaline soils in arid regions are often phosphorus-limited^50,51^.

### Microbial resource limitations and influencing factors

The study found that pH was negatively correlated with E_C:P_ and E_C:N_, likely because soil acidity and alkalinity directly affect the biochemical reaction rates of soil enzymes. Soil pH regulates microbial physiology by influencing the spatial conformation of enzymes, controlling both enzyme production and the binding of substrates^21^. Higher pH may inhibit the activity of the four enzymes, limiting increases in extracellular enzyme activity. Previous studies have also shown correlations between soil enzyme activity and pH^48,52,53^, supporting the findings of this study. RDA results indicated that soil depth and mining distance had a significant negative effect on vector length (VL) (Figs. 5 c,d). Previous research shows that soil moisture differences caused by spatial factors significantly influences enzyme activity, particularly in water-limited regions^54,55^. High soil moisture can enhance microbial substrate and enzyme diffusion rates, promoting microbial activity and function^56^. However, our study area, located in desert grasslands with limited precipitation, restricts soil moisture availability, reducing microbial substrate uptake and thereby inhibiting microbial growth and activity. In low-precipitation regions, competition between soil microbes and plants for carbon suppresses microbial biomass carbon accumulation^57^, further contributing to microbial carbon limitation.

AP is a key factor influencing ecoenzymatic stoichiometry (Fig. 5b). This may be due to the direct impact of phosphorus availability on the decomposition and cycling of carbon, nitrogen, and phosphorus in the soil, which indirectly affects the activity of enzymes involved in their metabolism. As a result, changes in ecoenzymatic stoichiometry occur. In phosphorus-limited areas, AP plays a crucial role as a nutrient and energy source for microbial growth, regulating microbial activity and influencing resource limitation^58^. Our study found that AP is positively correlated with carbon limitation but negatively correlated with phosphorus limitation (Fig. 5c), aligning with Wang et al.^59^ The soil nutrients had a strong negative effect on microbial biomass (Fig. 6a), Devi et al.^60^ also suggested that the soil nutrient content strongly influences the soil microbial biomass. RDA analysis showed that microbial biomass carbon (MBC) and nitrogen (MBN) were significantly and positively correlated with E_C:N_ and E_C:P_(Fig. 5a). The SEM results further supported this, revealing a positive effect of microbial biomass on ecoenzymatic stoichiometry (Fig. 6). This could be attributed to the poor soil conditions in the study area, where microbial biomass, as an essential source of nutrients, strongly influences soil enzyme activity^61^, and thus affects ecoenzymatic stoichiometry. Similarly, Acosta-Martinez and Harmel^62^ also reported a positive correlation between microbial biomass and enzyme activity.

The vector length of the soil ecoenzymatic stoichiometry gradually decreased with increasing soil depth in the three zones, indicating that the extent to which the soil microorganisms were C-limited decreased with increasing depth. The variation in the vector angle of the ecoenzymatic stoichiometry reveals that the soil microorganisms in the study area were mainly P-limited, and the extent of this limitation increased with increasing depth, consistent with our second hypothesis. Soil depth is also an important factor influencing ecoenzymatic stoichiometry and microbial resource limitation (Fig. 5b). Organic matter and nutrients are typically concentrated in the topsoil (0–10 cm), where plant debris and root systems are abundant, leading to higher microbial activity. Therefore, microbial biomass and activity are greater in shallow soil layers, resulting in higher enzyme activity. As soil depth increases, the amount of organic matter, such as plant root exudates and litter, decreases, leading to a decline in microbial numbers and enzyme secretion. This reduction in enzyme secretion causes a downward trend in ecoenzymatic stoichiometry. In deeper soil, competition for nutrients among microbes intensifies, which exacerbates P limitation. He et al.^63^also reported a significant positive correlation between soil depth and microbial P limitation. The distance from the mine drainage point influences ecoenzymatic stoichiometry and microbial resource limitation by affecting environmental factors and enzyme activity (Fig. 6a). In the zone closest to the discharge, known as the riparian area, mining water’s mobility and permeability result in higher water content and salinity. This area may also retain salt ions, such as SO_4_^2-^ and Cl^-^, from the mining water. As the distance from the discharge point increases, these effects diminish, leading to a negative impact of mining distance on environmental factors and enzyme activity.

It is worth noting that although the distance from mine drainage did not have a significant impact on microbial resource limitation, this does not mean that the degree of mine water pollution does not vary significantly with the distance from the drainage point. we believe that the absence of substantial changes in ecoenzymatic stoichiometry with distance does not imply that mine drainage pollution is spatially uniform. Our data show clear spatial variations in key pollutants: for instance, soil salinity is significantly higher in the littoral zone (0–20 m) than in the riparian (30–60 m) and upland (70–100 m) zones (Fig. 1), confirming a distance-dependent pollution gradient. The divergence in responses between enzyme activity and ecoenzymatic stoichiometry arises from their distinct regulatory mechanisms. Extracellular enzyme activity is highly sensitive to microenvironmental conditions such as soil moisture and salinity (Fig. 1). The littoral zone, closest to the drainage point, exhibits higher moisture and salinity, which promote microbial activity. Ecoenzymatic stoichiometry reflects microbial resource demand for C, N, and P1,2, which is primarily regulated by nutrient availability rather than pollutant concentration alone. In our study, mine drainage contains negligible phosphorus (0.04 mg/L) and thus fails to alleviate the inherent P limitation in desert grassland soils (sandy texture with poor P retention). This persistent P limitation dominates microbial metabolism across all distances, leading to relatively stable enzyme allocation ratios.

## Materials and methods

### Site description and experimental design

**Fig. 7:**
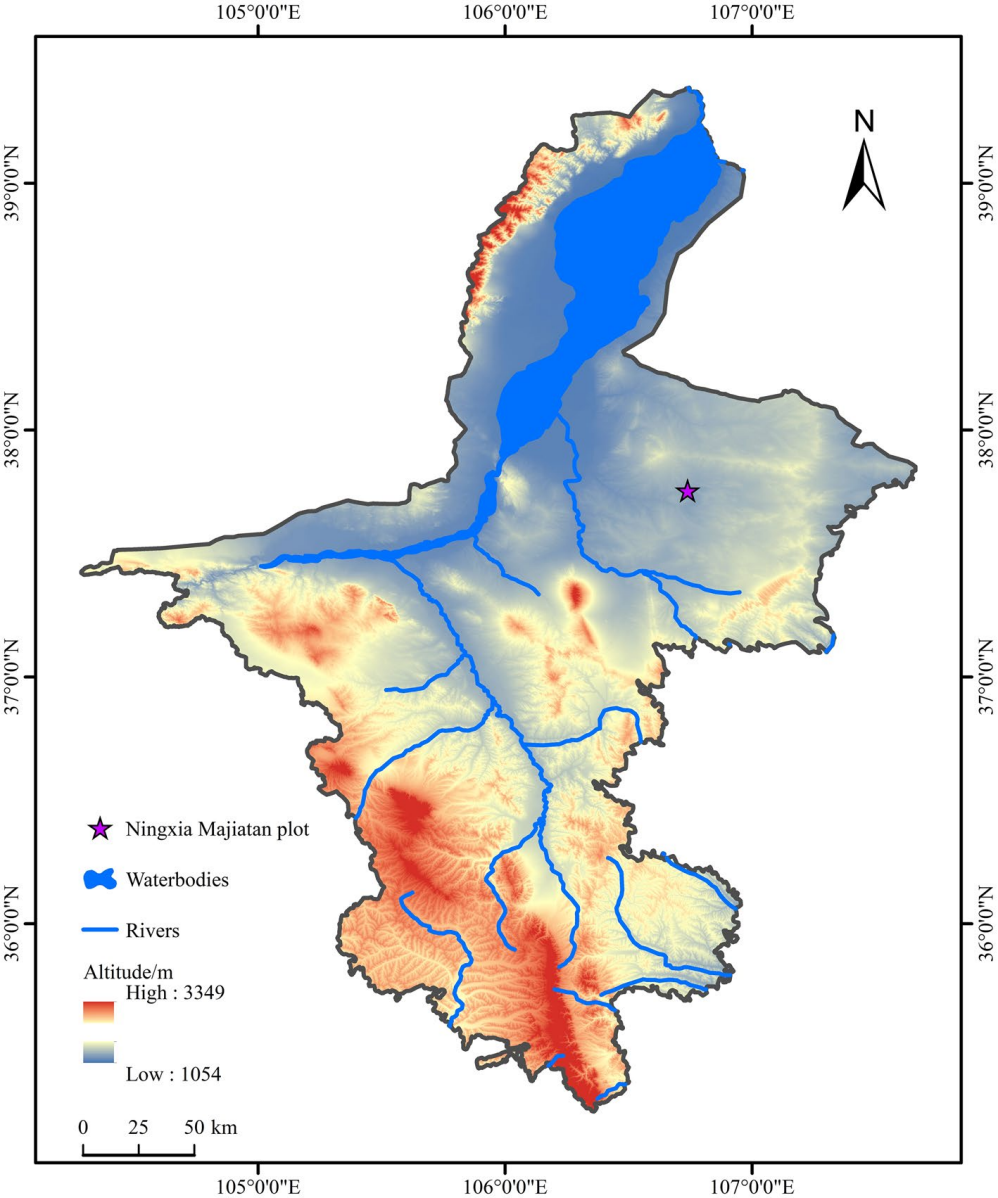
Map showing the geographic location of the sampling site. The map was created using ArcGIS 10.2 software (available at https://my.esri.com).

The study area is a desert grassland habitat used as a mine drainage site (37°45’N, 106°44’E; elevation 1300–1390 m) in Majiatan Town, Lingwu City, Ningxia Province, China (Fig. 7). Chemical analysis indicated low heavy metal concentrations in the mine water, below pollution levels. The water quality was characterized by a pH of 8.05 and concentrations of ammonia nitrogen (0.54 mg/L), total nitrogen (0.52 mg/L), total phosphorus (0.04 mg/L), SO_4_^2−^ (3840 mg/L), Cl^−^ (3435 mg/L), and total alkalinity (169 mg/L). The area experiences a mid-temperate arid climate with large diurnal temperature variations, frequent droughts, and low precipitation. The soil consists primarily of sandy and loose sandy loam, with low water and nutrient retention capacities. Vegetation in the area is dominated by halophytes and drought-resistant species like *Suaeda glauca*, *Halogeton glomeratus*, *Agropyron cristatum*, and *Artemisia desertorum*.

Sampling zones were established in a mine-water outlet area with no anthropogenic disturbance other than the discharge. Three zones were established at varying distances from the mine drainage point to represent different disturbance intensities: the littoral zone, the riparian zone and the upland zone. These zones reflect varying levels of disturbance caused by mine drainage. Six large plots (15 × 10 m) were established in each zone on flat terrain with uniform vegetation. A comprehensive analysis of the soil ecosystem was conducted through spatial (distance-based) and vertical (depth-based) sampling to evaluate the effects of mine drainage (Table 2).

**Table 2:** Soil sample distribution and analysis in different sampling zones.

Sampling Zone		littoral zone	riparian zone		upland zone
Distance from Mine Water Discharge (m)		0–20 m	30–60 m		70–100 m
Dominant Vegetation		A. cristatum	A. cristatum and Artemisia ordosica Krasch mix		Artemisia ordosica Krasch
Soil Depths Sampled (cm)		0–10 cm, 10–20 cm, 20-30cm			
Number of Large Plots (15 m × 10 m)		6	6		6
		Within each large plot, five smaller plots (1 × 1 m) were created using the five-point sampling method. The five samples from each depth in each zone were pooled and divided into two fractions			
Number of samples used for analysis	Soil enzyme activity	18	18		18
		These samples were stored in a labeled cryopreservation tube and kept at − 80°C for enzyme activity analysis			
	Soil physicochemical indicators	18	18		18
		These samples were air-dried and sieved for soil physicochemical indicators			
Total Number of Soil Samples		36	36		36

### Soil physicochemical properties and enzymes

Soil salinity was determined by employing the gravimetric method.Soil moisture content(SMC) was determined using the oven-drying method.The soil pH was measured in a suspension with a water-to-soil ratio of 2.5:1 using a PHS-3C pH meter (YouKe, Shanghai, China). The soil organic carbon (SOC) content was assessed by the potassium dichromate oxidation-external heating method. Total nitrogen (TN) content was determined using an ELEMENTAR elemental analyzer (Elementar Analysensysteme GmbH, Germany). Total phosphorus (TP) content was analyzed using the NaOH fusion-molybdenum antimony colorimetric method, while available phosphorus (AP) was extracted with NaHCO₃ and measured by the molybdenum antimony colorimetric method. Ammonium nitrogen (NH₄⁺-N) and nitrate nitrogen (NO₃⁻-N) concentrations were determined using a continuous flow analyzer^64^.

Microbial biomass carbon (MBC), microbial biomass nitrogen (MBN), and microbial biomass phosphorus (MBP) were quantified by the chloroform fumigation-extraction method^65,66^. Soil enzyme activities were measured using microplate fluorometry. The activities of β-N-acetylglucosaminidase (NAG), leucine aminopeptidase (LAP), β-1,4-glucosidase (BG), and alkaline phosphatase (ALP) were determined by fluorescence after reactions with their respective substrates: 4-MUB-N-acetyl-β-D-glucosaminide, L-leucine-7-amino-4-methylcoumarin, 4-MUB-β-D-glucoside, and 4-MUB-phosphate^67^ (Table S1).

### Microbial resource limitation

A stoichiometric analysis of soil enzyme activity can estimate potential resource limitations in soil microorganisms^68^. Four different groups of soil microbial resource limitation (N limitation, P limitation, C and P limitation, and N and P limitation) can be identified via plotting the ratio of (NAG + LAP) : ALP on the x-axis and the ratio of BG: (NAG + LAP) on the y-axis and denoting horizontal and vertical coordinates of 1 as baselines.

The enzyme vector model developed by Moorhead et al.^22,23^ was used to determine the nutrient limitation characteristics of the soil microorganisms. This model quantifies the microbial acquisition of C, N, and P simultaneously by plotting the allocation proportions of the enzyme activities to generate vector lengths and angles. The Vector model’s calculation formula is as follows:

1 $$Vector Length = \sqrt{{x}^{2}+{y}^{2}}$$

2 $$Vector \, Angle \, = \, Degrees \, \left( {Atan2 \, \left( {x, \, y} \right)} \right)$$

where x represents the relative activities of C vs. P acquiring enzymes (ln BG/ ln ALP), and y represents the relative activities of C vs. N acquiring enzymes [ln BG /ln (NAG + LAP)]. The results of the vector model indicate that the length of the vector represents the relative C limitation, and a longer length indicates greater relative C limitation. A vector angle of greater than 45° indicates microbial P limitation, while an angle of less than 45° indicates N limitation^22^.

### Data analysis

Before analysis, normal distribution and homogeneity were tested for all data. Data collection and analyses were performed through Microsoft Excel 2021 and SPSS software (version 26, IBM). The soil physicochemical factors, microbial biomass, and extracellular enzyme activity were analyzed using one-way and two-way ANOVA analysis^69^. Bar graphs and box plots were generated with Prism 9. The redundancy analyses (RDA) were created or adopted through R software (version 4.3.3) with the “ggplot2”and “vegan” packages^70–74^. An envfit analysis (envfit function used with 999 permutations, “vegan” package) was associated with the last RDA for identifying the significant environmental factors that effect the enzyme acctivity, ecoenzymatic stoichiometry and microbial resource limitation. SmartPLS is a software with a graphical user interface for variance-based structural equation modeling (SEM) using the partial least squares (PLS) path modeling method.This study will employ partial least squares structural equation modeling (PLS-SEM) to analyze both the direct and indirect effects of environmental factors on microbial resource limitation^75^.

In the partial least squares structural equation modeling (PLS-SEM) analysis, standardized total effects (including direct and indirect effects) were calculated to quantify the magnitude and direction of relationships between variables. All observed variables were first standardized using z-score normalization (i.e., transforming values to have a mean of 0 and a standard deviation of 1) to eliminate the influence of different measurement units. This standardization ensures that the effect sizes of variables with distinct scales are comparable.The standardized total effect of an exogenous variable on an endogenous variable (e.g., carbon limitation or phosphorus limitation) was computed as the sum of its standardized direct effect and all standardized indirect effects (mediated through other variables in the model). The significance of total effects was assessed using bootstrapping with 5000 resamples, and effects with p < 0.05 were considered statistically significant.

## Conclusions

This study analyzed the spatial changes in soil physicochemical properties, microbial biomass, extracellular enzyme activity, and stoichiometry under the influence of mine drainage. The riparian zone experienced significant nutrient loss, soil quality degradation, and severe salinization due to mine drainage. The effects of the discharge on microbial biomass carbon, nitrogen, and phosphorus were varied. Mine water significantly increased the activity of four extracellular enzymes, with the highest levels observed in the riparian zone, and clear differences among soil layers. Activities of β-glucosidase (BG), N-acetylglucosaminidase (NAG), and leucine aminopeptidase (LAP) decreased with increasing soil depth, while alkaline phosphatase (ALP) showed the opposite trend. Key factors influencing soil extracellular ecoenzymatic stoichiometry and microbial resource limitation under mine drainage were identified. Redundancy analysis (RDA) and structural equation modeling clarified the complex interactions, revealing soil depth and mining distance as key drivers. These factors indirectly affected shifts in carbon (C) and phosphorus (P) limitations by altering soil nutrients, environmental factors, enzyme activity, and econzymatic stoichiometry. Phosphorus limitation intensified with increasing soil depth. Overall, these findings offer deeper insights into how spatially varying mine drainage reshapes soil ecoenzymatic stoichiometry and microbial resource limitations, guiding future ecological management strategies.

## Supplementary Information

Supplementary Information 1.

Supplementary Information 2.

## Data Availability

Data is provided within the supplementary information files.
