# Supplementary material for: Mine drainage leads to the reshaping of the spatial patterns of soil extracellular econzymatic stoichiometry and microbial resource limitation
Source: Sci Rep. 2025 Nov 27;15:42844. doi: 10.1038/s41598-025-20858-1 (PMC12669616; doi:10.1038/s41598-025-20858-1)
Supplement: Supplementary file 2 — Supplementary Information 2. [file 41598_2025_20858_MOESM2_ESM.docx]

**Mine Drainage leads to the reshaping of the spatial patterns of soil extracellular enzyme stoichiometry and microbial resource limitation**

Ruoshi Ma ^a^, Jieru KONG ^a^, Hongxia MOU ^b^, Bingru LIU ^a,^*

^a^ School of Biological Science and Engineering, North Minzu University, Yinchuan 750021, China

^b^ School of Ecological Environment, Ningxia University. Yinchuan, 750021, China

* **Corresponding author**

Bingru LIU ( Email: [bingru.liu@163.com)](mailto:flinc629@hotmail.com)Abstract)

**Table S1 Enzyme activity analysis substrate**

| Enzyme | Substrate | Enzyme commission numbers |
| --- | --- | --- |
| β-1,4- glucosidase（BG） | 4-MUB-b-D- glucoside | 3.2.1.21 |
| alkaline phosphatase（ALP） | 4-MUB- organic phosphate | 3.1.3.2 |
| β-1,4-N-acetyl-glucosaminidase （NAG） | 4-MUB-N-acetyl-b-D-Acylglucosamine | 3.1.6.1 |
| leucine aminopeptidase (LAP) | L-leucine-7-amino-4-methylcoumarin | 3.5.2.10 |

|  | A | | B | | A*B | |
| --- | --- | --- | --- | --- | --- | --- |
|  | *F*-value | *p* value | *F*-value | *p* value | *F*-value | *p* value |
| pH | **39.411^***^** | <0.001 | 1.297 | 0.283 | 0.878 | 0.485 |
| salinity | **407.784^***^** | <0.001 | **4.695**^*^ | 0.014 | **4.912**^**^ | 0.0002 |
| SOC | **82.362^***^** | <0.001 | 2.885 | 0.066 | 2.393 | 0.065 |
| NO_3_^-^-N | 1.358 | 0.268 | 2.964 | 0.062 | 0.713 | 0.588 |
| NH_4_^+^-N | 1.469 | 0.241 | 1.525 | 0.229 | 1.128 | 0.356 |
| TN | **12.433^***^** | <0.001 | 1.23 | 0.302 | 1.076 | 0.38 |
| TP | **11.908^***^** | <0.001 | **3.871** | 0.028 | 1.263 | 0.299 |
| AP | 3.008 | 0.059 | **20.237^***^** | <0.001 | 0.56 | 0.693 |
| SMC | **880.202^***^** | <0.001 | **50.517^***^** | <0.001 | 0.82 | 0.52 |
| MBC | **6.246^**^** | 0.004 | **8.59^***^** | <0.001 | **5.996^***^** | <0.001 |
| MBN | **43.614^***^** | <0.001 | **10.129^***^** | <0.001 | **3.564*** | 0.013 |
| MBP | **8.37^***^** | <0.001 | 0 | 1 | 1.565 | 0.2 |
| BG | **27.024^***^** | <0.001 | **44.99^***^** | <0.001 | **4.779^**^** | 0.003 |
| ALP | **103.192^***^** | <0.001 | **92.52^***^** | <0.001 | **14.306^***^** | <0.001 |
| NAG | **74.053^***^** | <0.001 | **8.751^***^** | <0.001 | 1.534 | 0.209 |
| LAP | **14.374^***^** | <0.001 | **5.728^**^** | 0.006 | 0.595 | 0.668 |

**Table S2**  Results (F and p values) of two-way ANOVA for the effects of horizontal coastal zone and soil depth on soil, microorganisms, and extracellular enzymes and stoichiometry. Significant effects are highlighted in bold (p < 0.05).

Note: A, mining distance; B, soil depth; A × B, the interaction between the mining distance and soil depth. *, **, *** indicate significant differences at p < 0.05, p < 0.01, and p < 0.001, respectively.
